# Space-Weather Utilities for Research and Forecasting (SURF): A Tool for Investigating Hydrodynamic Aspects of Solar Wind and Coronal Mass Ejection Expansion and Evolution

**DOI:** 10.1007/s11207-026-02738-7

**Published:** 2026-09-28

**Authors:** Mathew J. Owens, Luke A. Barnard

**Affiliations:** Department of Meteorology, University of Reading, Earley Gate, PO Box 243, Reading, RG6 6BB UK

## Abstract

We introduce Space-weather Utilities for Research and Forecasting (SURF), a flexible modelling framework for reconstructing and forecasting large-scale solar wind structure. SURF incorporates both the reduced hydrodynamic “HUXt” model and a newly developed one-dimensional, hydrodynamic solver, “hydro.”. The new solver introduces more physically consistent compression effects, while retaining the computational efficiency required for ensemble forecasting, uncertainty quantification, and large parametric studies. SURF-hydro solves the 1D Euler equations in spherical geometry using finite-volume methods with Riemann solvers and second-order spatial reconstruction. It is shown to accurately reproduce an analytical solution to pressure-driven expansion of a uniform spherical solar wind. Using 30 years of near-Earth OMNI observations, we derive empirical relations between speed, density, and temperature at 1 AU and map these back to 0.1 AU to provide non-equilibrium inner-boundary conditions for SURF-hydro (and for use with other solar wind models). This approach produces more realistic solar wind speed, density and temperature values and variability at 1 AU than simulations based on equilibrium thermodynamic relations at 0.1 AU. While magnetic pressure and tension forces are not included, SURF-hydro is also shown to reproduce the key features of coronal mass ejection (CME) expansion and evolution. For one particular example of a structured solar wind, sensitivity tests demonstrate that CME density and temperature at 0.1 AU alter 1 AU transit times and arrival speeds by 15 – 20%, highlighting a potential under-explored source of forecast uncertainty. SURF-hydro therefore provides a computationally efficient bridge between idealised models and full 3D magnetohydrodynamic simulations, enabling systematic investigation of boundary condition assumptions and CME parameter sensitivity for both research and operational space-weather forecasting.

## Introduction

Reconstruction of the state of the large-scale solar wind underpins a huge range of scientific and space weather forecasting applications. The standard methodology is to couple coronal and solar wind (heliospheric) models, typically with an interface around 0.1 AU, where the solar wind flow is already super-magnetosonic. There are now a wide range of three-dimensional magnetohydrodynamic (3DMHD) solar wind models, such as Enlil (Odstrcil and Pizzo 1999), EUHFORIA (Pomoell and Poedts 2018), SWMF (Toth et al. 2005), SUSANOO (Shiota and Kataoka 2016), HelioMAS (Riley, Linker, and Mikic 2001), ICARUS (Baratashvili et al. 2025), HelioCubed (Pogorelov et al. 2024) and GAMERA (Merkin et al. 2016; Zhang et al. 2019). These models accurately capture the large-scale dynamics of a magnetised fluid flow. But there are many applications where the computational expense and code complexity of 3DMHD becomes prohibitive. These include large ensembles of runs for uncertainty quantification (Owens and Riley 2017) and data assimilation (Barnard et al. 2023), large-scale parametric studies (Gyeltshen et al. 2026), long-duration runs for planetary studies (O’Donoghue et al. 2025), development of adjoint models for data assimilation (Lang and Owens 2019), etc. In such situations it is useful to sacrifice a small amount of physical fidelity for greatly increased computational efficiency and code simplicity. Highly idealised and empirical models can be both extremely rapid and useful, particularly for specific applications in forecasting (e.g. Hinterreiter et al. 2021). But there is also a middle ground, retaining the fluid approach with discretised numerical solutions, but employing what is best referred to as a “reduced-physics” approximation. The heliospheric upwind extrapolation model with time dependence (HUXt; Owens et al. 2020; Barnard and Owens 2022) is one such reduced-physics model that has found use for both forecasting (https://swxforecastlab.org/forecasts.html) and a diverse range of scientific applications (e.g. O’Donoghue et al. 2025; Watson et al. 2025; Chi et al. 2023; Yu et al. 2023).

HUXt treats the solar wind as a one-dimensional, reduced hydrodynamic advection problem. It dynamically solves for the radial flow speed and the heliospheric magnetic field polarity, which is treated as a passively advecting property with the flow. The 1D approximation is well justified as the solar wind flow is observed to be highly radial in most situations (e.g. Scherer et al. 2001; Owens and Cargill 2004), and even complex three dimensional structures like coronal mass ejections (CMEs) behave as quasi-one-dimensional structures – at least to first order – owing to their rapid spherical expansion (Owens, Lockwood, and Barnard 2017; Owens 2020a). The hydrodynamic approximation can also be justified with simple order-of-magnitude arguments, with the 1-AU solar wind displaying dynamic flow pressure around two orders of magnitude higher than either the thermal or magnetic pressures in most circumstances. The lack of physics-based compressibility is perhaps the hardest to justify. While HUXt has been shown to produce flow fields in very close agreement (within 5%) with 3DMHD for the same boundary conditions, the key differences are found at compression fronts at stream interaction regions (Owens et al. 2020). We here seek to introduce more realistic flow dynamics through compression. To this end, we introduce a compressible hydrodynamic solver – “hydro” – that does not sacrifice too much of the computationally efficiency that makes HUXt a useful forecast and research tool.

A significant amount of open-source supporting infrastructure has been built up around the HUXt model. The HUXt codebase now contains easy-to-use capability for driving HUXt with semi-empirical (Arge et al. 2003) and full MHD (Linker et al. 1999) coronal model output, as well as from coronal tomography (Bunting et al. 2024) and cone-model CMEs (Zhao, Plunkett, and Liu 2002), and boundary conditions derived directly from in situ observations (Owens et al. 2026a). Support exists for both inner heliosphere ensemble (Owens and Riley 2017) and outer heliosphere science (O’Donoghue et al. 2025), and for both steady-state and time-dependent (Owens, Barnard, and Arge 2024) ambient solar wind modelling. HUXt can readily be run in pseudo 2- or 3-dimensions, with field-line tracing, time series and CME arrival times at any specified body, and with an array of visualisation options. We seek to exploit this pre-existing modelling framework for the new hydro model and other future developments. For this reason, we refer to the general modelling framework as “SURF”; Space-weather Utilities for Research and Forecasting. HUXt and hydro are modelling options within SURF. This framework also permits future developments to be easily incorporated, such as one-dimensional MHD solvers (e.g. Tao et al. 2005; Riley and Ben-Nun 2022), or true two- or three-dimensional hydrodynamic solvers.

Section 2 reviews the physical equations we seek to solve with the new ‘SURF-hydro’ model, while Section 3 describes the specific numerical implementation used. Section 4 outlines the analytical ‘Parker nozzle’ model of a spherically expanding fluid which we use to validate the numerical SURF-hydro solutions. The results are split across two sections: Section 5 details how the ambient solar wind boundary conditions are determined and the resulting model solutions at 1 AU, whereas Section 6 investigates the application to transient CME structures. Section 7 summaries the results and possible future avenues for research.

## Physical Basis

The evolution of the solar wind flow can be approximated by the equations of mass, momentum and energy conservation for a hydrodynamic flow. We here follow the basic framework outlined in the ‘pyro’ python library of hydrodynamic examples (Harpole et al. 2019; Zingale 2014). However, we develop a stand-alone, pure python solver, fully integrated within the SURF framework, rather than produce a ‘wrapper’ for the existing pyro code.

Ignoring gravity and viscous stress, we seek to solve:

1 $$ \text{conservation of mass: } \frac{\partial \rho}{\partial t} + \nabla \cdot (\rho \mathbf{v}) = 0, $$

2 $$ \text{conservation of momentum: } \frac{\partial (\rho \mathbf{v})}{\partial t} + \nabla \cdot (\rho \mathbf{v}\mathbf{v}) + \nabla p = 0, $$

3 $$ \text{and conservation of energy: } \frac{\partial (\rho E)}{\partial t} + \nabla \cdot ( \mathbf{v} E ) + \nabla \cdot (p \mathbf{v}) = 0, $$

where ρ is the mass density, $\mathbf{v}$ is the fluid velocity, p is the fluid pressure and E is the total fluid energy density, given by:

4 $$ E = \frac{1}{2} \rho \mathbf{v}^{2} + E_{INT}, $$

where $E_{INT}$ is the internal energy density of the gas. For an ideal gas:

5 $$ E_{INT} = \frac{p}{\gamma - 1} , $$

where γ is the polytropic index. Early solar wind models assumed an ideal monatomic gas and set γ to 5/3 (Hundhausen and Gentry 1969). However, this generally results in the temperature dropping off more rapidly with distance that is observed (Totten, Freeman, and Arya 1995). For this reason γ is commonly taken to be around 1.5 for solar wind modelling (Jacobs and Poedts 2011). In SURF, γ can be specified by the user, with the change propagated consistently through all the boundary specification methods outlined below. However, a default value of 1.5 is used and a full assessment of how this affects the solar wind properties as a function of radial distance will be the focus of future work.

Considering only the radial (r) direction in spherical geometry, $\mathbf{v}$ is reduced to the radial speed, v, and the 1D Euler equation becomes:

6 $$ \frac{\partial \mathbf{U}}{\partial t} + \frac{1}{r^{2}} \frac{\partial (r^{2} \mathbf{F})}{\partial r} = \mathbf{S} , $$

where the conserved variables, $\mathbf{U}$, are:

7 $$ \mathbf{U} = \begin{bmatrix} \rho \\ \rho v \\ \rho E \end{bmatrix} , $$

and the fluxes, $\mathbf{F}$, are:

8 $$ \mathbf{F} = \begin{bmatrix} \rho v \\ \rho v^{2} + p \\ v(E + p) \end{bmatrix} . $$

$\mathbf{S}$ is the geometric source vector. In Cartesian coordinates, $\mathbf{S} = 0$. However, in spherical coordinates, there is a geometric source of momentum arising from the fact that the surface area of a sphere grows as $1/r^{2}$ (Harpole et al. 2019; Zingale 2014). This results in a geometric pressure gradient. Therefore:

9 $$ \mathbf{S} = \begin{bmatrix} 0 \\ \frac{2p}{r} \\ 0 \end{bmatrix} . $$

## Numerical Implementation

In order to solve the physical equations in Section 2, they are discretized in radial distance, r, and time, t, using a finite volume method. Thus a conserved variable at coordinates (r,t) is written as:

10 $$ \mathbf{U}(r, t) \rightarrow \mathbf{U}^{n}_{i} = \mathbf{U}(r_{i}, t_{n}) $$

where $r_{i}$ is the radial distance of the centre of the ith grid cell, and $t_{n}$ is the nth time step. We solve on a fixed, uniform radial grid, with grid cell spacings of Δr. We refer to the interface between cell i and i+1 as i+1/2. Time steps are denoted by n, with spacing between consecutive time steps as Δt. This time step is variable to ensure stability (see discussion below). Note that the SURF-hydro output, however, is linearly interpolated onto a fixed, uniform time grid to ensure compatibility with the existing plotting and analysis codebase.

As each grid cell is part of a spherical shell, the area at each cell interface is $A_{i \pm 1/2} = 4 \pi r_{i \pm 1/2}^{2}$. The evolution of a conserved quantity at grid cell i is then given by:

11 $$ \frac{d \mathbf{U}}{dt} = \mathcal{L} (\mathbf{U}) $$

where ℒ is the spatial discretization operator:

12 $$ \mathcal{L} (\mathbf{U}) = - \frac{1}{V_{i}} \left ( r_{i+1/2}^{2} \mathbf{F}_{i+1/2} - r_{i-1/2}^{2} \mathbf{F}_{i-1/2} \right ) + \mathbf{S}_{i} $$

and $V_{i}$ is the volume of the shell at distance $r_{i}$:

13 $$ V_{i} = \frac{1}{3} ( r_{i+1/2}^{3} - r_{i-1/2}^{3}). $$

As shown in Equation 9, the geometric source terms are zero for mass and energy. For momentum, however, spherical geometry results in a net component of the pressure force acting in the radial direction. Thus the source term is given by:

14 $$ \mathbf{S_{i}} = \begin{bmatrix} 0 \\ p_{i} \frac{A_{i+1/2} - A_{i-1/2}}{v_{i}} \\ 0 \end{bmatrix} $$

In order to compute the fluxes through the inner and outer cell boundaries, we must first must compute $\mathbf{U}$ either side of the cell interfaces, rather than just the average values at the cell centres. Two reconstruction approaches are used. Like the original HUXt code, one method is a first-order approach, the piecewise constant method (PCM). In PCM, the value either side of the interface is simply assumed to be the value at the cell centre. So at interface i+1/2 (the outer boundary of cell i and inner boundary of cell i+1) the state at the inner edge of the interface is given by $\mathbf{U_{INNER}} = \mathbf{U_{i}}$, while the outer edge is given by $\mathbf{U_{OUTER}}= \mathbf{U_{i+1}}$.

However, the default method in SURF-hydro is to use a second-order solution, the Piecewise Linear Method (PLM). This uses the linear gradient in $\mathbf{U}$, such that at the i+1/2 interface, $\mathbf{U_{INNER}} = \mathbf{U_{i + 1/2}} \Delta \mathbf{U_{i}}$ and $\mathbf{U_{OUTER}} = \mathbf{U_{i+1}} - 1/2 \Delta \mathbf{U}_{i+1}$. This retains sharper features, such as shocks, but requires a gradient limiter. We use the monotonized central limiter (Van Leer 1977) to prevent spurious oscillations while maintaining second-order accuracy away from extrema.

Next, for both reconstruction methods, we must solve for the discontinuities across cells. We use a Harten-Lax-van Leer-Contact solver (HLLC Toro 2009; Toro, Spruce, and Speares 1994) that assumes the solution consists of two waves (inner and outer) and a contact discontinuity in between. Either side of the contact discontinuity – and between the inner and outer cell interfaces – are two intermediate ‘star states’, $\mathbf{U_{*INNER}}$ and $\mathbf{U_{*OUTER}}$. The pressure and velocity are equal in these two regions, but the density and energy may be different. The HLLC solver evaluates the wave speeds and determines which region ($\mathbf{U_{i-1/2}}$, $\mathbf{U_{*INNER}}$, $\mathbf{U_{*OUTER}}$, $\mathbf{U_{i+1/2}}$) is sampled at the interface, then returns the corresponding flux.

The final step is the time update. For the PCM reconstruction, we use the first-order forward Euler time integration, where:

15 $$ \mathbf{U^{n+1}} = \mathbf{U^{n}} + \Delta t \mathcal{L}( \mathbf{U^{n}}) $$

For the PLM reconstruction, it is necessary to use the second-order Runge-Kutta method. Here we first compute the predictor state, $\mathbf{U^{p}}$, which is the same as the first-order solution:

16 $$ \mathbf{U^{p}} = \mathbf{U^{n}} + \Delta t \mathcal{L}(\mathbf{U^{n}}) $$

and then to compute the second-order correction:

17 $$ \mathbf{U^{n+1}} = \mathbf{U^{n}} + \frac{\Delta t}{2} \left [ \mathcal{L}(\mathbf{U^{n}}) + \mathcal{L}(\mathbf{U^{p}}) \right ] $$

For both PCM and PCL, the time step must be chosen to meet the Courant-Friedrichs-Lewy (CFL) stability condition, such that:

18 $$ \Delta t = C \min _{i} \left ( \frac{\Delta r}{|v_{i}| + c_{i}} \right ) $$

where $c_{i} = \sqrt{\gamma p_{i}/ \rho _{i}}$ is the local sound speed and C is a constant to ensure stability. To balance computational efficiency and stability we use default values of C=0.8 for PCM and 0.6 for PLM.

As in HUXt, the user can specify the radial grid, with the time step being automatically adjusted to match the associated CFL conditions. A default grid size of 1.5 solar radii ($r_{S}$) is used. Two- and three dimensional HUXt runs involve running a series of independent SURF-HUXt and SURF-hydro one-dimensional models. Thus while longitudinal and latitudinal grid spacing can also be specified, this has no direct numerical or stability implications. By default, 128 longitudinal grid cells are used, and 45 latitudinal cells. All plots and analysis in this study use the default grids.

## Validation

In order to test the numerical implementations of SURF-hydro described above, we compare with an analytical solution for a steady-state solar wind expansion in diverging spherical geometry. This differs from the classic Parker solar wind solution (Parker 1958) in that it ignores gravity and allows temperature to vary adiabatically. Thus it involves the exact same physics as SURF-hydro.

This solution treats the solar wind acceleration as a one-dimensional isentropic flow through a nozzle with cross-sectional area $A(r) \propto r^{2}$. It can then be shown (Anderson 2021) that the temperature, density and flow speed are given by:

19 $$\begin{aligned} T(r) &= T_{0} \left [ \frac{1 + \frac{\gamma -1}{2}M_{0}^{2}}{1 + \frac{\gamma -1}{2}M(r)^{2}} \right ] \end{aligned}$$

20 $$\begin{aligned} v(r) &= v_{0} \frac{M(r)}{M_{0}} \sqrt{\frac{T(r)}{T_{0}}} \end{aligned}$$

21 $$\begin{aligned} \rho (r) &= \rho _{0} \frac{M_{0}}{M(r)} \sqrt{\frac{T_{0}}{T(r)}} \left (\frac{r_{0}}{r}\right )^{2} \end{aligned}$$

where $T_{0}$, $v_{0}$ and $\rho _{0}$ are the temperature, speed and mass density at the inner boundary distance $r_{0}$ and M(r)=v(r)/c(r) is the local Mach number.

This analytical solution exactly conserves mass flux ($\rho v r^{2} = \mathrm{const}$) and provides a benchmark for validating SURF-hydro in smooth, shock-free flows (Toro 2009).

**Figure 1:**
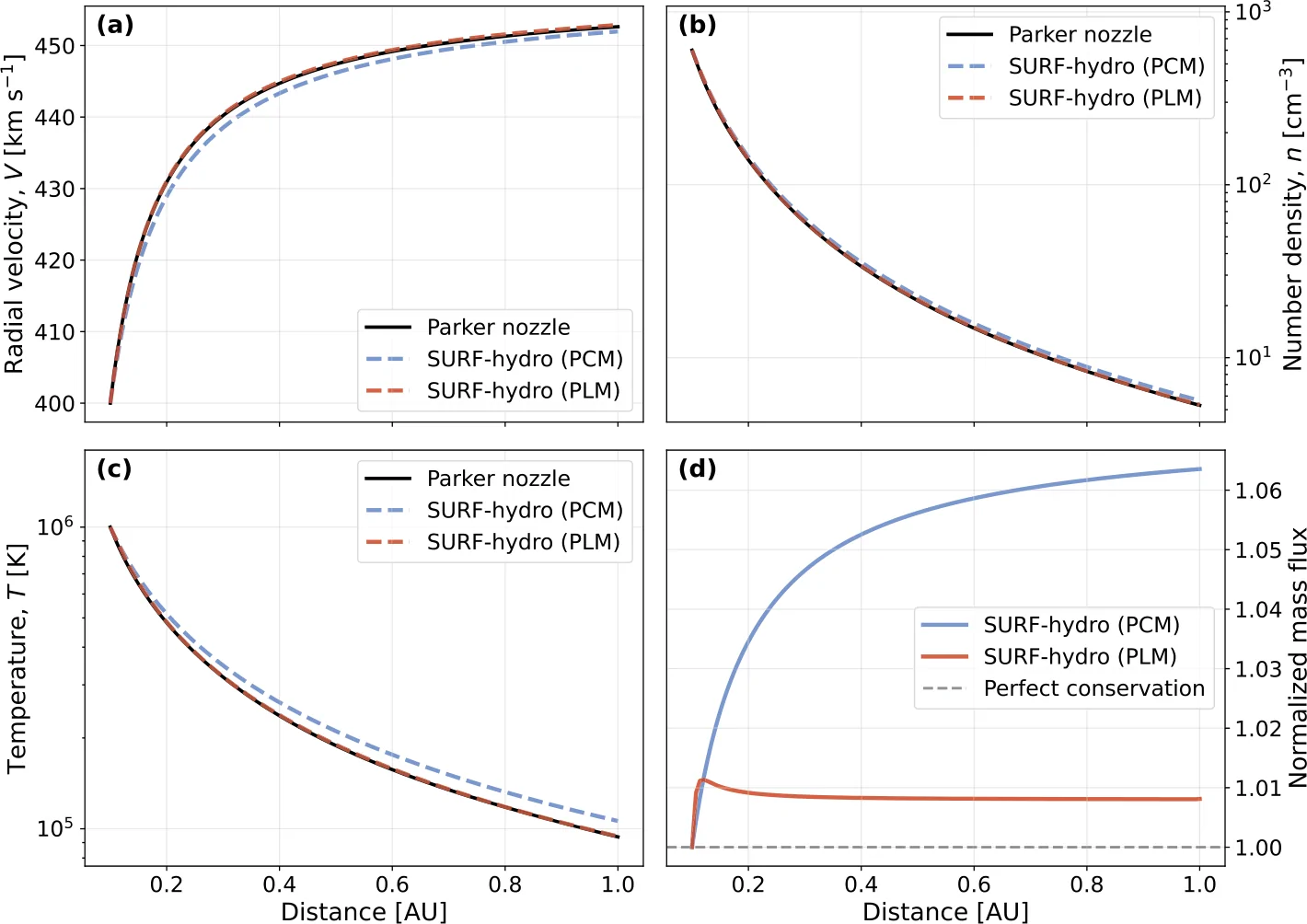
Comparison of SURF-hydro and analytical solar wind solutions from 0.1 to 1 AU (21.5 to 215 Rs). (a) Solar wind speed, v, as a function of heliocentric distance. The analytical solution is shown as a solid black line, with SURF-hydro PCM and PLM solutions shown as blue and red dashed lines, respectively. (b) number density, $n_{P}$, assuming a proton plasma, (c) temperature, T (d) fractional change in total mass.

Figure 1 shows comparisons of the SURF-hydro solutions with the analytical steady-state solar wind solution. All models use inner boundaries at 0.1 AU and inner boundary conditions of $v = 400\text{ km}\,\mathrm{s}^{-1}$, T = 1 MK and a mass density computed from assuming a proton plasma of number density $n_{P} = 600\text{ cm}^{-3}$. For the analytical model, these values result in 1 AU properties of $v = 453\text{ km}\,\mathrm{s}^{-1}$, T = 94,000 K and $n_{P} = 5\text{ cm}^{-3}$, which are fairly typical values (e.g. Owens 2020b). For the SURF-hydro solutions, the model solutions are shown after the standard spin-up time of 5 days, to allow propagation of the inner boundary conditions through the whole 1-AU domain.

By eye, the SURF-hydro-PLM solution to v is nearly identical to the analytical benchmark solution. Over the whole domain, the error in v is 0.04%, in $n_{P}$ is 0.89%, and in T is 0.85%. For SURF-hydro-PCM, the deviation from the analytical benchmark solution is visible in the plots, particularly for temperature. Specifically, over the whole domain, the error in v is 0.40%, in $n_{P}$ is 5.63% and in T is 11.17%. Perhaps more important is the conservation of mass in the simulation. For SURF-hydro-PCM, the mass flux deviates from the inner boundary condition by 5.5% at 1 AU and is still continuing to gradually rise even at the outer boundary. This could be reduced by increasing the spatial and temporal resolution, but that would come with additional computation cost and PCM would still have difficulty with capturing shocks. By contrast, the second-order scheme of SURF-hydro-PLM conserves mass to within 1%. Furthermore, even this small deviation is due entirely to the second-order scheme needing to interpolate across the inner boundary. Within the main computational domain, mass is conserved to machine accuracy.

For PCM, the 5-day run for a single longitude takes 0.03 seconds on a standard desktop CPU, while the PLM run takes 0.10 seconds. This compares with the original HUXt runtime of the same setup of around 0.02 seconds. Thus the second-order solver does add significant – approximately x5 – computational overhead compared to HUXt, but SURF-hydro is still rapid enough for most of the original HUXt use cases. The PLM solver is used through the remainder of this paper. However, PCM is still included as an option within SURF for specific situations where large numbers of ambient solar wind solutions are required, and the computation time is particularly critical.

## Ambient Solar Wind

The solar wind acceleration is prescribed as an analytical function in SURF-HUXt. This empirical relation is intended to mimic the spherical pressure gradient (Riley and Lionello 2011). For SURF-hydro, this process arises naturally, meaning solar wind acceleration is sensitive to the choice of temperature and (to a lesser extent) density at the inner boundary. The value of γ also has a small effect.

**Figure 2:**
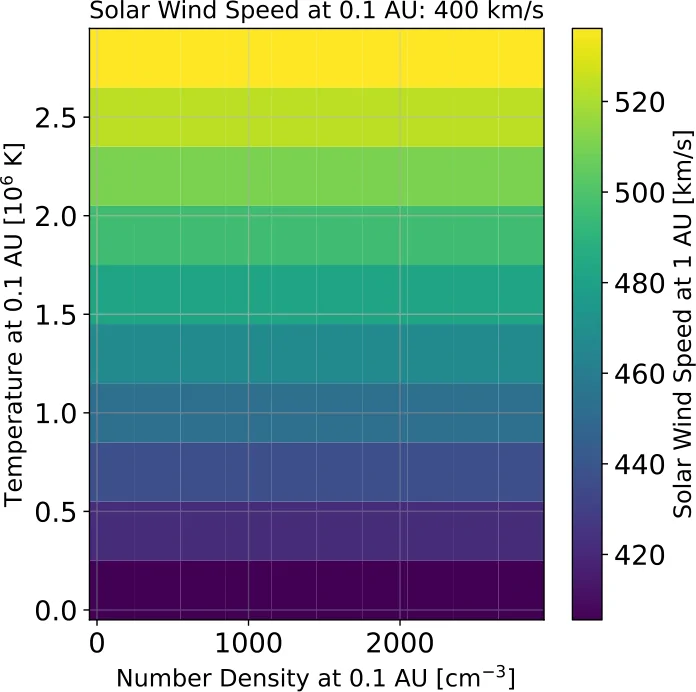
Solar wind speed at 1 AU for a range of proton density and temperatures at 0.1 AU. A uniform solar wind speed at 0.1 AU of 400 km s^−1^ is used throughout.

Figure 2 uses SURF-hydro to show how v at 1 AU varies for different T and $n_{P}$ at 0.1 AU for γ=1.5. A uniform v at 0.1 AU of 400 km s^−1^ is used throughout. T at 0.1 AU has a strong effect on the resulting solar wind acceleration. A temperature of 1 MK at 0.1 AU produces about a 10% increase in solar wind speed by 1 AU, whilst a 3 MK corona results in around a 30% increase. Conversely, solar wind density at 0.1 AU does not affect solar wind acceleration, despite contributing to the solar wind thermal pressure term. This is because the momentum is affected by pressure gradient with radius, which is unaffected by a change in the magnitude of the density. This is also evident from the Parker nozzle solution too, wherein the solar wind speed at a given difference depends only on the initial temperature and initial speed, not the density. Note, however, that once there is structure in the solar wind speed at 0.1 AU, the density will begin to affect dynamics, as it can then produce radial pressure gradients. This is shown in Section 6. As fast wind is typically less dense than slow wind, solar wind structure can affect the mean asymptotic solar wind speeds at 1 AU.

It is therefore important to accurately determine not only speed at 0.1 AU, but also temperature and density. While full MHD solutions of the corona can in principle directly provide this information (Perri et al. 2023), in practice these values often show insufficient variability. Instead, it is typical for only the radial magnetic field from corona models to be directly passed to solar wind models. Even for coronal MHD models, it is common for the radial solar wind speed at 0.1 AU to be determined from empirical relations to the coronal magnetic field configuration (Linker et al. 2016; Riley, Linker, and Mikic 2001). Thus it is also necessary to estimate $n_{P}$ and T at the inner boundary on the basis of the inner-boundary v. Typically some form of equilibrium state is assumed at the solar wind model inner boundary. A range of approaches is used; constant mass flux over the 0.1-AU sphere (Detman et al. 2006), constant kinetic energy flux (Odstrcil and Pizzo 1999; Pomoell and Poedts 2018) or constant momentum flux (Riley et al. 2011) are invoked to infer $n_{P}$ from v. Then constant thermal pressure over the 0.1-AU sphere is used to determine T. For use with interplanetary scintillation (IPS) derived solar wind speeds, there have been attempts to use purely empirical relations between $n_{P}$ and T with v, which do not explicitly impose flux or pressure balance (Hayashi, Tokumaru, and Fujiki 2016). However, these are typically applied much further out into the heliosphere (∼50−60 r_S_), where most solar wind acceleration has already ceased (e.g. see Figure 1a). Shiota et al. (2014) also used empirical relations derived from *Helios* observations to define $n_{P}$ and T at the inner boundary, and produced reasonable speeds in near-Earth space. However, they did not consider the accuracy of the resulting density and temperature at 1 AU.

In general, there has not been systematic validation of these assumptions across a large dataset, largely due to the computational cost of such endeavours. It is also likely (see analysis below) that such approaches have been primarily tuned to match solar wind speed in near-Earth space, rather than density and temperature, as that is the more immediate space-weather concern.

In future work, SURF-hydro will be used to assess the implications of all the equilibrium approaches over an extended time interval. Here, however, we demonstrate a simple non-equilibrium approach that performs well in reproducing solar wind speed, density and temperature at 1 AU.

**Figure 3:**
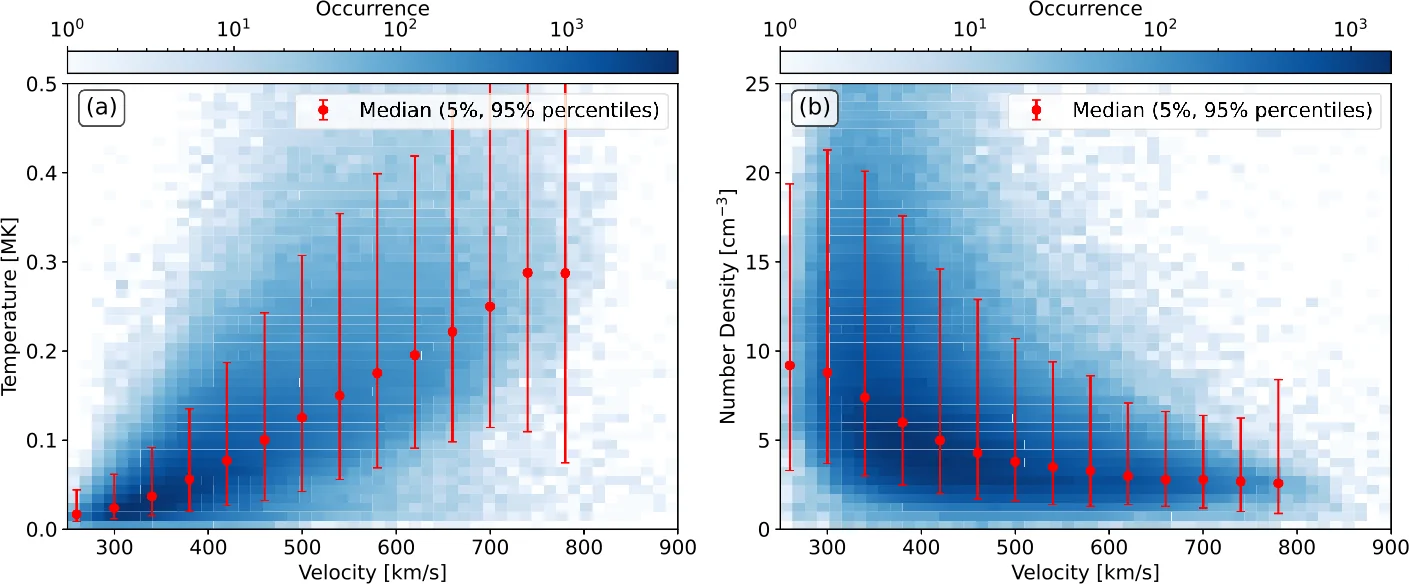
OMNI observations of solar wind parameters at 1 AU over the period 1995 – 2026 at 1-hour resolution. ICMEs have been removed. Panels show: (a) proton temperature, T, against proton radial velocity, v; (b) Proton number density, $n_{P}$, against v. The colour scale shows 2D histograms of occurrence on a log scale. The red dots show the median values in uniform v bins, with red lines spanning the 5- to 95 percentiles.

Figure 3 shows an analysis of near-Earth solar wind data in order to characterise the relations of density and temperature with radial speed at 1 AU. Using OMNI data for the period of near-contiguous observations (1995 to 2026) at 1-hour resolution, we first remove all periods identified as interplanetary CMEs (ICMEs) using the updated list from Richardson and Cane (2010). Two dimensional occurrence histograms are shown in Figures 3a and b. The data are then binned in v and the median values of density and temperature computed in each bin. These are listed in Table 1. Note that these are values at 1 AU, whereas we require relations between $n_{P}$, T and v at the SURF-hydro inner boundary radius (typically 0.1 AU).

**Table 1:** Table of median density and temperature for given solar wind speeds at 1 AU based on the OMNI dataset.

$v_{1\,\mathrm{AU}}$ (km/s)	$n_{1\,\mathrm{AU}}$ (cm^−3^)	$T_{1\,\mathrm{AU}}$
220	9.90	9.37e+03
260	9.20	1.71e+04
300	8.80	2.41e+04
340	7.40	3.73e+04
380	6.00	5.61e+04
420	5.00	7.71e+04
460	4.30	1.00e+05
500	3.80	1.26e+05
540	3.50	1.50e+05
580	3.30	1.75e+05
620	3.00	1.96e+05
660	2.80	2.22e+05
700	2.80	2.50e+05
740	2.70	2.88e+05
780	2.60	2.87e+05

Each set of v, $n_{P}$ and T values at 1 AU (as shown in Table 1) is then used with Equations 19 to 21 to compute the 0.1 AU equivalents. These back-mapped values are shown in Table 2 as $v_{0.1\,\mathrm{AU}}$, $n_{0.1\,\mathrm{AU}}$ and $T_{0.1\,\mathrm{AU}}$. Linear interpolation of this look-up table is used to compute $n_{P}$ and T at the inner boundary for any given value of $v_{0.1\,\mathrm{AU}}$. This look-up table approach avoids loss of fidelity that would result from fitting an analytical function to the data and allows for easy updates to the relations resulting from more sophisticated analysis of the observational data sets in the future.

**Table 2:** Table of solar wind speeds at 1 AU, with equivalent speeds and median densities and temperatures at 0.1 AU assuming γ=1.5. These are based on backmapping the values in Table 1 using the analytical functions in Equations 19 to 21.

$v_{1\,\mathrm{AU}}$ (km/s)	$v_{0.1\,\mathrm{AU}}$ (km/s)	$n_{0.1\,\mathrm{AU}}$ (cm^−3^)	$T_{0.1\,\mathrm{AU}}$ (K)
220	210	1037.0	9.59e+04
260	244	978.7	1.76e+05
300	281	940.1	2.49e+05
340	313	802.9	3.89e+05
380	343	663.9	5.91e+05
420	374	561.8	8.17e+05
460	404	489.1	1.07e+06
500	435	436.4	1.35e+06
540	468	403.7	1.61e+06
580	502	381.5	1.89e+06
620	539	345.3	2.10e+06
660	573	322.3	2.38e+06
700	608	322.5	2.68e+06
740	639	312.7	3.10e+06
780	686	295.6	3.06e+06

**Figure 4:**
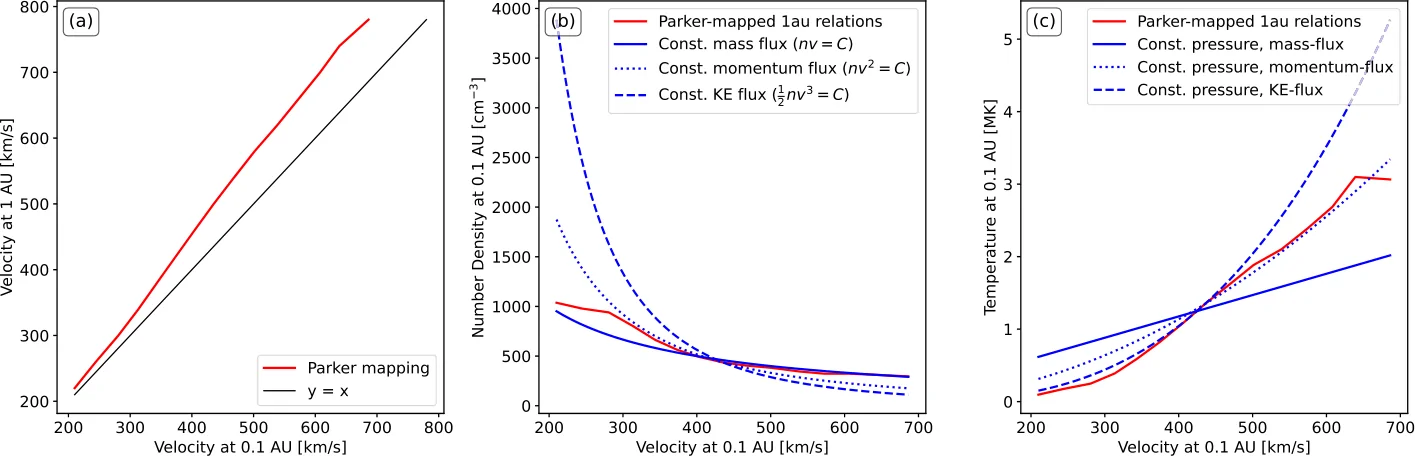
Relations used to construct the inner boundary conditions for solar wind models. (a) The red line shows relation between solar wind speed at 1 AU – such as is provided by the WSA model – and 0.1 AU, assuming the 1-AU relations with density and temperature, and using the Parker nozzle solution to solar wind acceleration. (b) Relations between solar wind speed and density at 0.1 AU. The SURF-hydro method – based on observed 1-AU empirical relations – is shown in red. For comparison, assumptions of constant mass flux, constant kinetic energy flux and constant momentum flux are shown by solid, dashed and dotted blue lines, respectively. (c) Relations between speed and temperature at 0.1 AU. The SURF-hydro method is shown in red. Solid, dashed and dotted blue lines shown the assumption of constant thermal pressure, using density from constant mass flux, constant kinetic energy flux and constant momentum flux, respectively.

Figure 4a shows the relation between 1-AU and 0.1-AU speeds, calculated assuming the observed 1-AU relations between speed with density and temperature, and applying the analytical Parker solution. Owing to the non-linear relations with density and temperature, the adjustment to speed is also non-linear, ranging from below 5% at low speeds, to around 15% at higher speeds. We suggest that this is the correction that should be applied to WSA coronal model speeds for use as 0.1 AU inner boundary conditions with dynamical solar wind models such as Enlil, ICARUS and EUHFORIA.

Figures 4b and c show the relation of speed to density and temperature, respectively, at 0.1 AU. The SURF-hydro relations shown in red are based on the observed 1-AU relations from OMNI data, backmapped using the Parker nozzle solution. Other commonly used assumptions of constant mass flux, constant momentum flux and constant kinetic energy flux are shown for comparison (constants are chosen to match the SURF densities and temperatures at around 400 km/s). Compared to the backmapped OMNI relations, constant kinetic energy flux tends to produce too much variation in density and temperature. Constant momentum flux produces too high density at low speeds, but broadly similar variations in temperature (though with approximately a factor 2 overestimate at low speeds). Conversely, assuming constant mass flux produces similar trends in density, but underestimates the temperature variation. The backmapped OMNI relations thus offer a means to avoid introducing biases associated with the various equilibrium boundary condition assumptions.

Computation of values at a 0.1-AU inner boundary is shown purely as a typical example. In practice, SURF uses the relations at 1 AU (Table 1) and the specified value of γ to compute bespoke look-up tables at the specific inner boundary distance required for each SURF-hydro run, which varies for different observational input sources and use cases. As with many of the boundary condition construction methods, these relations could be used with any solar wind model, not just the SURF models.

**Figure 5:**
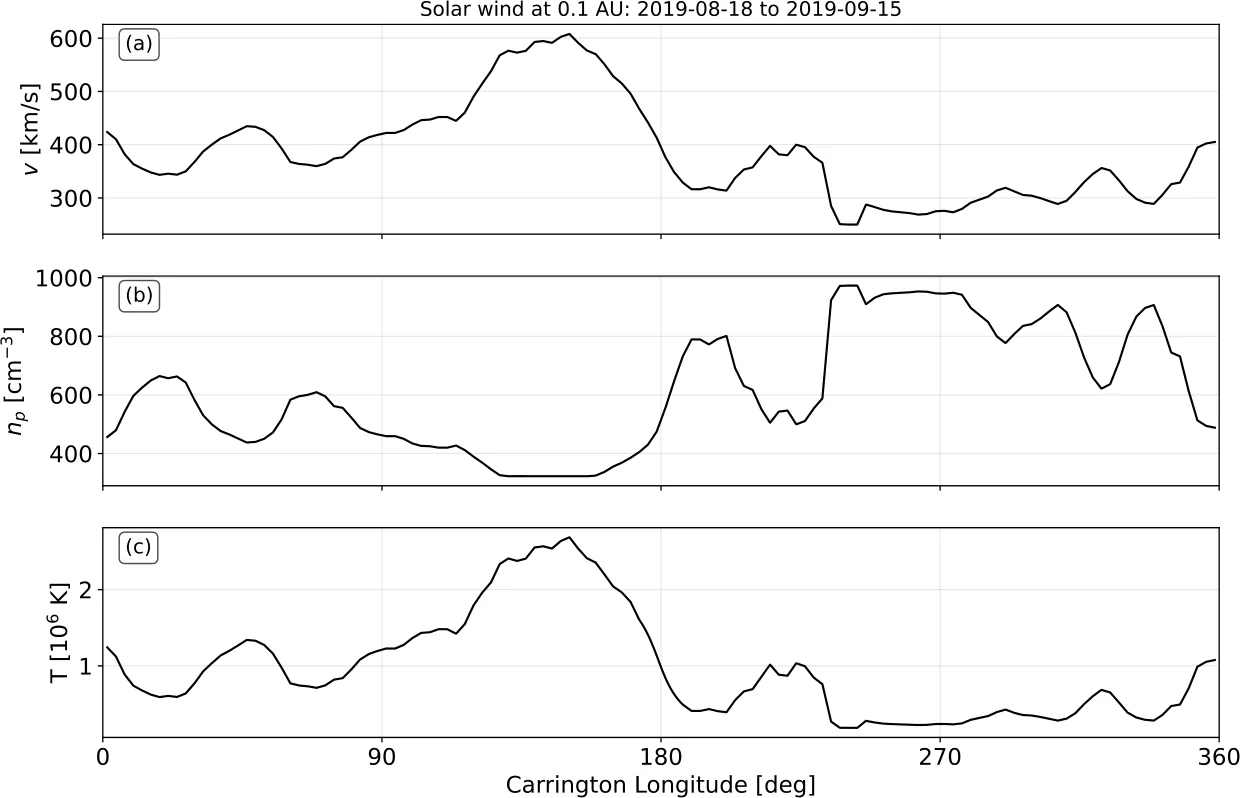
Solar wind properties at 0.1 AU as a function of Carrington longitude. (a) Solar wind speed at 0.1 AU, $v_{0.1\,\mathrm{AU}}$, generated by ‘backmapping’ near-Earth OMNI solar wind observations for the period 2019-8-18 to 2019-9-15, following the methodology of Owens et al. (2026a). (b) Proton number density at 0.1 AU derived from $v_{0.1\,\mathrm{AU}}$ and the relations in Table 2. (c) Proton temperature at 0.1 AU, derived from $v_{0.1\,\mathrm{AU}}$ and the relations in Table 2.

In order to demonstrate how well these empirical relations work, we produce an example solar wind hindcast with SURF-hydro. OMNI near-Earth solar wind observations for 2019-8-18 to 2019-9-15 are first used to construct v as a function of Carrington longitude at 0.1 AU, following the methodology of Owens et al. (2026a). Similar approaches of using in situ observations to set model inner boundary conditions have been used by Li et al. (2016), Tasnim, Cairns, and Wheatland (2018) and Biondo et al. (2021). The back-mapped solar wind speed is shown as Figure 5a. Estimates of $n_{P}$ and T at 0.1 AU are then derived from the empirical relations in Table 2. These are shown in Figures 5b and c. This provides a complete set of boundary conditions for SURF-hydro.

**Figure 6:**
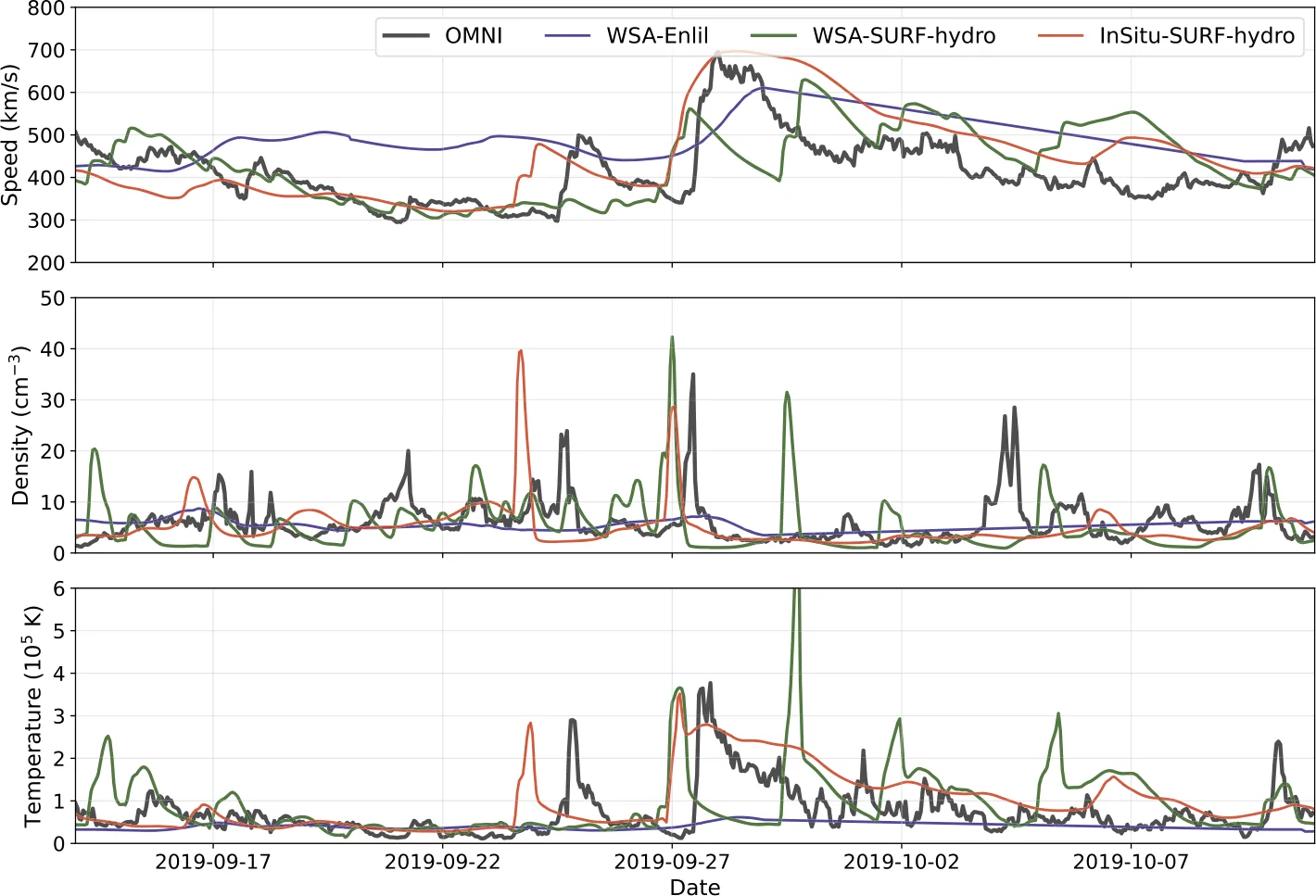
Solar wind hindcasts made on 2019-9-15 for the next 27 days. Black lines show OMNI observations. Red lines SURF-hydro driven by backmapped in situ observations. For comparison, a series of 5-day forecasts from the Space Weather Prediction Center’s operational WSA-Enlil forecasts are shown in blue. And daily-updated WSA-SURF hindcasts are shown in green. (a) Solar wind speed at 1 AU, (b) proton number density at 1 AU, and (c) proton temperature at 1 AU.

The 0.1 AU boundary conditions were then used to drive SURF-hydro forward in time for a further 27 days from 2019-9-10. The model has no access to any observations after 2019-9-10, so this can be considered a hindcast. The results are shown in Figure 6.

For this example interval, the InSitu-SURF-hydro hindcast (in red) reproduces the general solar wind speed structure in near-Earth space very well, as the previous 27 days had also been similar. Of course, this is not always the case, as shown in Owens et al. (2026b); this interval was specifically chosen as it is a representative example of recurrent solar wind and we here seek to determine how well density and temperature can be reconstructed when the large-scale solar wind speed structure is broadly correct. The results are very promising. The base-level proton number density of around 5 cm^−3^ is well captured, with stream interaction regions (SIRs) producing solar wind compressions and density spikes lasting hours with magnitudes of 10 s cm^−3^. There is a similar picture for temperature, with both observed and modelled proton temperature ranging from around $5\times 10^{4}\text{ K}$ in very slow wind, to $5\times 10^{5}\text{ K}$ in the compressed solar wind at stream interaction regions.

The blue lines in Figure 6 show WSA-Enlil forecasts for the same period, obtained from the archive at the Community Coordinated Modelling Center (CCMC): https://iswa.ccmc.gsfc.nasa.gov/iswa_data_tree/model/heliosphere/wsa-enlil-cone/. The WSA-Enlil time series is constructed from four separate 5-day forecasts, so the forecast lead time is generally much lower that the SURF runs, which varies from 0 to 27 days over the interval shown. There is some evidence that the observed pattern in v is present in the WSA-Enlil solution, but the dynamic range is much reduced. There is almost no variation in $n_{P}$, while T is systematically an order of magnitude too low. Thus the SURF-hydro solution is providing much more accurate and useful information at this time. Of course, this is not a model issue; Enlil contains a more accurate representation of solar wind physics than SURF-hydro. But the solar wind solution at 1-AU is primarily determined by the model boundary conditions (Heinemann et al. 2025), not the model physics.

To demonstrate this, we produce a third hindcast using the same WSA maps, but with SURF-hydro. As in WSA-Enlil, WSA speeds are reduced from their ‘high’ 1 AU values to provide suitable 0.1 AU values. We use the first two columns in Table 2 for this purpose. Unlike WSA-Enlil, however, the density and temperature at 0.1 AU are set using the empirical, non-equilibrium boundary conditions. The result is shown in green. Using this different treatment of the boundary conditions, the variability in speed, density and temperature is improved compared to the equilibrium boundary conditions of WSA-Enlil. We note that for the stream interaction region on 2019-9-29, WSA-SURF-hydro produces a temperature spike around twice as large as any observed in this interval. This may be due to the 1D nature of the solution; deflection of flow away from the radial is not possible. This could likely be mitigated with suitable smoothing of sharp gradients in the inputs at the SURF inner boundary.

**Figure 7:**
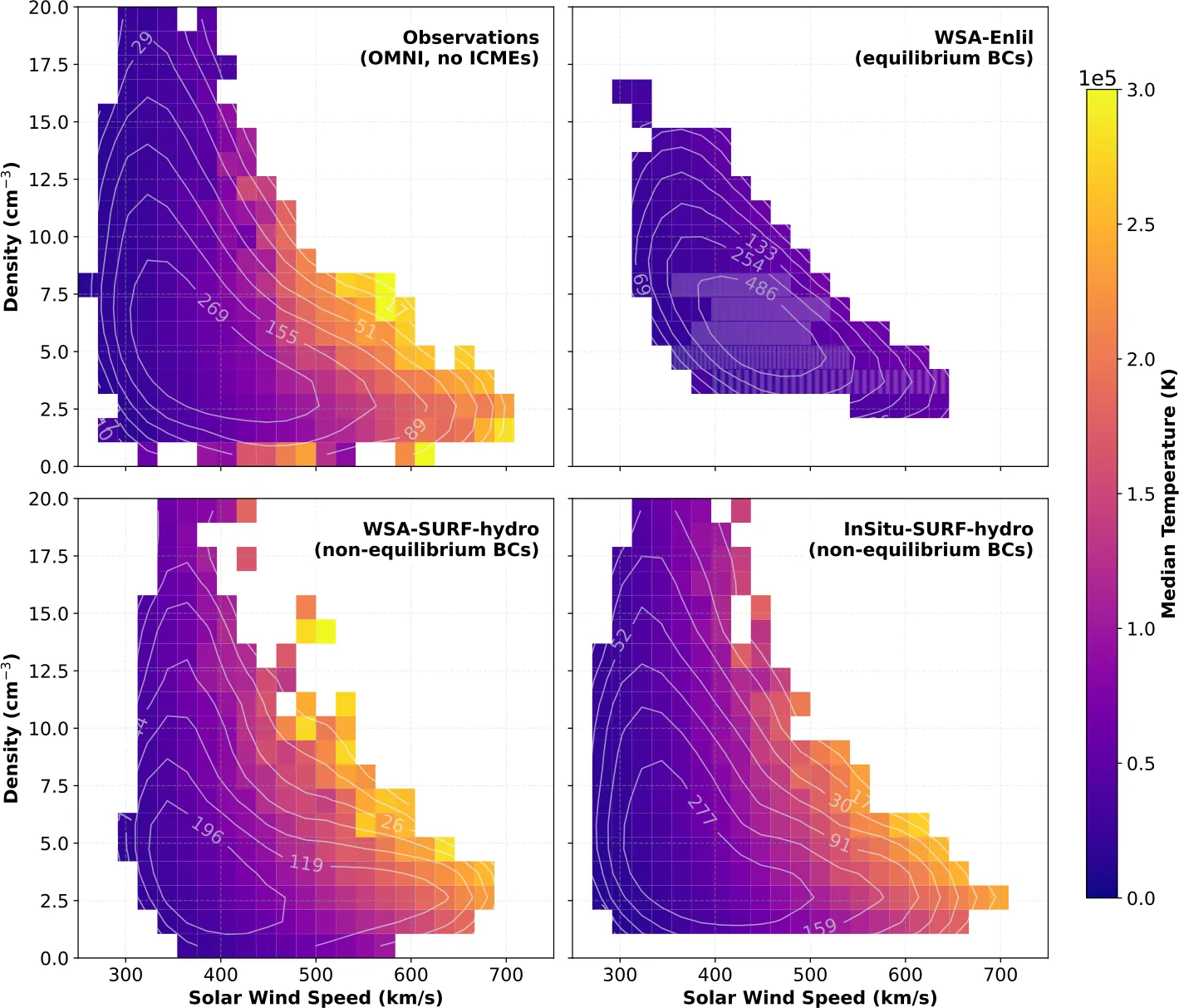
Solar wind conditions at 1 AU over the period 2019 – 2023 at 1-hour resolution for (top-left) OMNI in situ observations, (top right) WSA-Enlil, (bottom-left) WSA-SURF-hydro and (bottom-right) InSitu-SURF-hydro. For each bin, mean density is shown as a function of mean solar wind speed, with mean temperature indicated by the colour scale. Only points with at least 10 values are shown. The white contours show occurrence frequency.

We now expand this single hindcast to a 4 year interval. The top-left panel of Figure 7 shows 1-hour OMNI near-Earth solar wind observations over 2019 – 2023, with periods identified as ICMEs by the Richardson and Cane (2010) catalogue removed. The data have been binned by density and speed. Only parameter space containing at least 10 values is shown. Occurrence contours are shown in white, while the colour bar shows the median proton temperature. In general, slow wind is more dense and cooler than fast wind. However, slow wind also shows a much greater range of densities than fast wind. Similar plots have been shown by Rivera et al. (2025).

The top-right hand panel shows 1 AU properties for WSA-Enlil solutions over the same period. There are a number of notable difference between WSA-Enlil and OMNI observations. Firstly, the range of speeds in WSA-Enlil is reduced compared to observations. This could be a result of under-dispersed speeds in the WSA solution, or pre-processing of the WSA speeds prior to use as an Enlil boundary condition. This lack of variability in speed would then lead to the observed lack of variability in density too, as there is reduced opportunity to form strong stream interaction regions. However, it is clear that the problem is more fundamental than that, as the WSA-Enlil temperatures are also far too low, even allowing for the reduced range of speeds produced by WSA-Enlil. This must be an issue with specifying the inner-boundary temperatures, suggesting that the equilibrium assumption employed in WSA-Enlil is limiting the accuracy of the resulting simulations.

The bottom-left panel shows WSA-SURF-hydro, using non-equilibrium density and temperature relations at 0.1 AU. The range of speeds at 1 AU is slightly reduced compared to the OMNI observations, but not to the same extent as WSA-Enlil. The range of densities and temperatures in WSA-SURF-hydro is also in better agreement with the observations, as are the general trends between speed and density/temperature. The major difference is at intermediate solar wind speeds (450 to 550 km s^−1^), where WSA-SURF produces higher densities and temperatures than observed. This is likely the result of WSA producing too many fast streams and hence compression regions (Owens et al. 2026b).

The bottom-right panel of Figure 7 shows the InSitu-SURF-hydro solution. As the speeds at 0.1 AU are specified by backmapping the OMNI observed speeds, the fact that InSitu-SURF-hydro matches the observed range of speeds has been explicitly built into the methodology. What is important, however, is that the 0.1-AU densities and temperatures that are derived using the empirical relations in Table 2 then produce the correct range of densities and temperatures at 1 AU. It also shows broadly similar correlations to those observed, with fast wind being less dense and hotter than slow wind.

We therefore suggest that WSA-Enlil (and other coupled corona-heliosphere models) could be significantly improved by employing similar empirical density and temperature relations, rather than the current equilibrium assumptions that are used.

It should be noted that these examples only consider one point in the heliosphere; near-Earth space. Thus the radial acceleration could still be systematically biassed. It is important to test against inner heliosphere observations, such as provided by *Parker Solar Probe* and *Solar Orbiter*. This will provide a much stronger constraint on the solar wind acceleration (e.g. Badman et al. 2025; Rivera et al. 2025, 2024).

## Coronal Mass Ejections

The primary purpose of a space-weather modelling system is to represent the time-dependent flows associated with coronal mass ejections (CMEs). The standard operational forecasting approach is to characterise the kinematics of CMEs – speed, direction, angular width – by fitting a simple geometric model to white-light coronagraph observations (Zhao, Plunkett, and Liu 2002). A perturbation at the solar wind model inner boundary is then introduced with the same basic kinematic properties (Odstrcil, Riley, and Zhao 2004). This localised, transient perturbation can be a simple speed perturbation, or also involve changes to the solar wind density and temperature at 0.1 AU. There has not been a great deal of systematic research into exactly how best to make these perturbations. For example, the WSA-Enlil operational forecasting system inserts a perturbation with the coronagraph-estimated CME speed, a typical ambient solar wind temperature at 0.1 AU and four times a typical ambient solar wind density at 0.1 AU (Zheng et al. 2013; Taktakishvili et al. 2009). Uncertainty in the density and temperature of CMEs is not something that is currently considered in any ensemble forecasting (Mays et al. 2015), primarily because the parameter space is large and multiple runs of 3DMHD models are computationally expensive. Very limited sensitivity analysis of these properties has been performed for a single event (Falkenberg et al. 2010), though this has primarily focussed on further increasing the CME perturbation to be hotter and denser than the ambient solar wind conditions at 0.1 AU. The rationale behind this is that the cone-model representation of CMEs neglects the strong internal magnetic field of CMEs, and thus increased thermal pressure is required to make up for the lack of internal magnetic pressure (see also Verbeke et al. 2022). This over-dense approximation to a CME was first used by Gosling et al. (1998).

**Figure 8:**
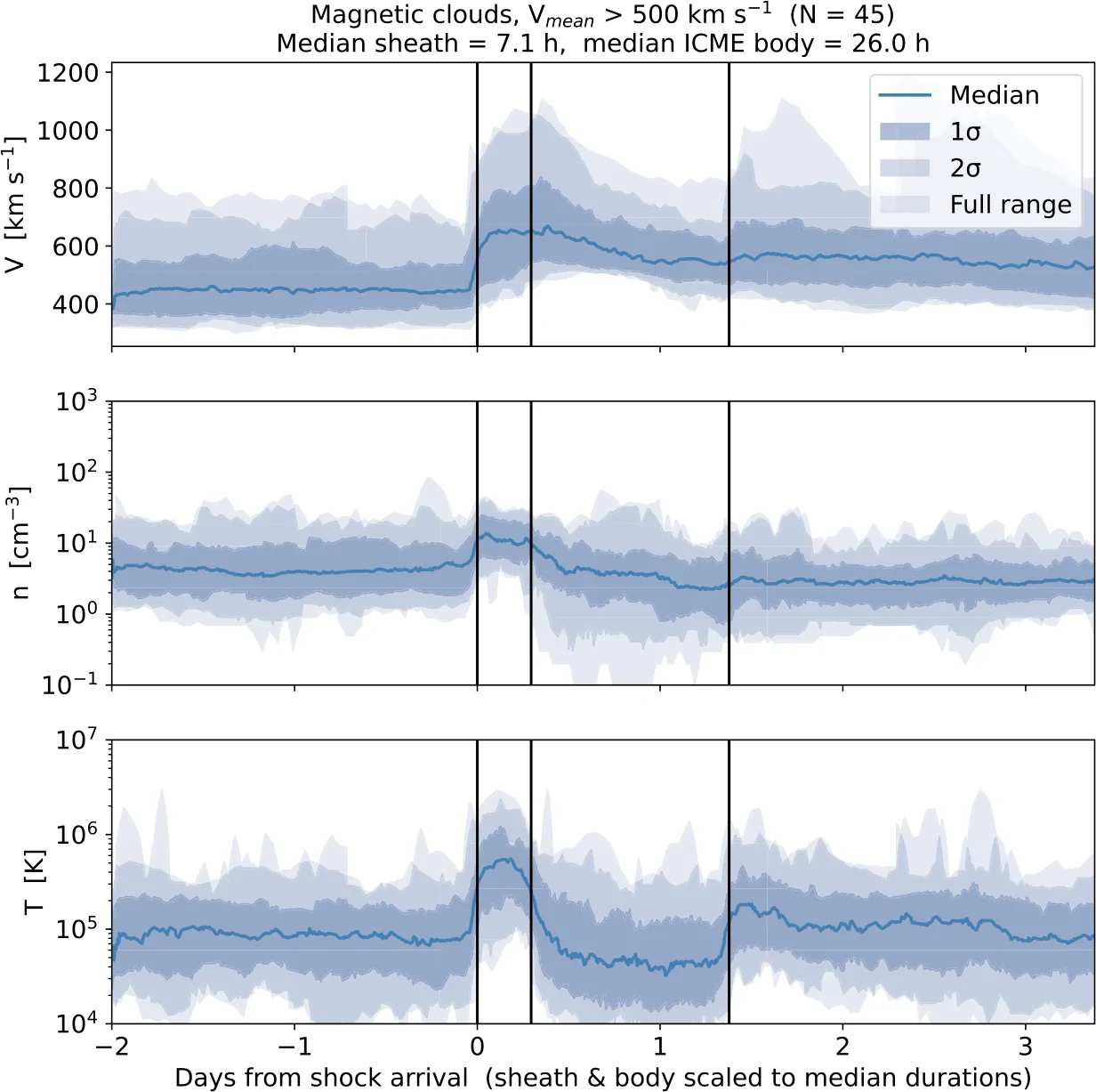
Super-posed epoch analysis of OMNI near-Earth solar wind data for all fast magnetic clouds between 1994 and 2025 (N = 45). Top: solar wind speed, middle: proton density, bottom: temperature. The median sheath duration for these events is 7.1 hours, and the median ICME body duration is 26 hours. All event durations have been normalised to these values to show typical variations. The vertical lines show the shock, ICME leading and trailing edges identified in the Richardson and Cane (2010) catalogue. The median value at each time is shown by the blue line, with lighter shades showing 1-σ, 2-σ and the full range of values.

However, ICMEs at 1 AU are known to be very low proton density and proton temperature (Richardson and Cane 2010) compared to ambient solar wind conditions. To demonstrate this, Figure 8 shows a super-posed epoch analysis of OMNI observations for all magnetic clouds in the Richardson and Cane (2010) catalogue with an average speed greater than 500 km s^−1^. We choose the fast events so that the sheath region ahead of the ICME can be included too. These magnetic clouds have the strongest internal magnetic fields and so should be the most challenging to reproduce in SURF-hydro. The median sheath duration for these events is 7.1 hours, while the median ICME duration is 26 hours. All event time series are normalised to these durations. For this ‘typical’ event, the sheath region is characterised by fast, dense and hot solar wind. The ICME body is characterised by a declining speed profile, and lower density and temperature than the surrounding ambient solar wind. This density and temperature reduction partly arises from adiabatic expansion during transit from 0.1 to 1 AU (Démoulin and Dasso 2009; Agarwal and Mishra 2024). But there is also evidence that CMEs close to the Sun have already undergone significant expansion and cooling (Romeo et al. 2023; Davies et al. 2024). Therefore it seems plausible that CMEs at 0.1 AU should be represented as low density and temperature perturbations at 0.1 AU, rather than the current over-dense perturbations used operationally.

SURF-hydro is technically well suited to investigating this problem, as parameter space can be rapidly sampled. The magnetic pressure is neglected, but as discussed above, even in 3DMHD models CMEs are generally treated as hydrodynamic structures. Thus the core physics of adiabatic expansion and cooling is still well represented. Indeed, 1D hydrodynamic models have strong heritage in successful interpretation of CME dynamics and expansion (e.g. Gosling et al. 1998).

**Figure 9:**
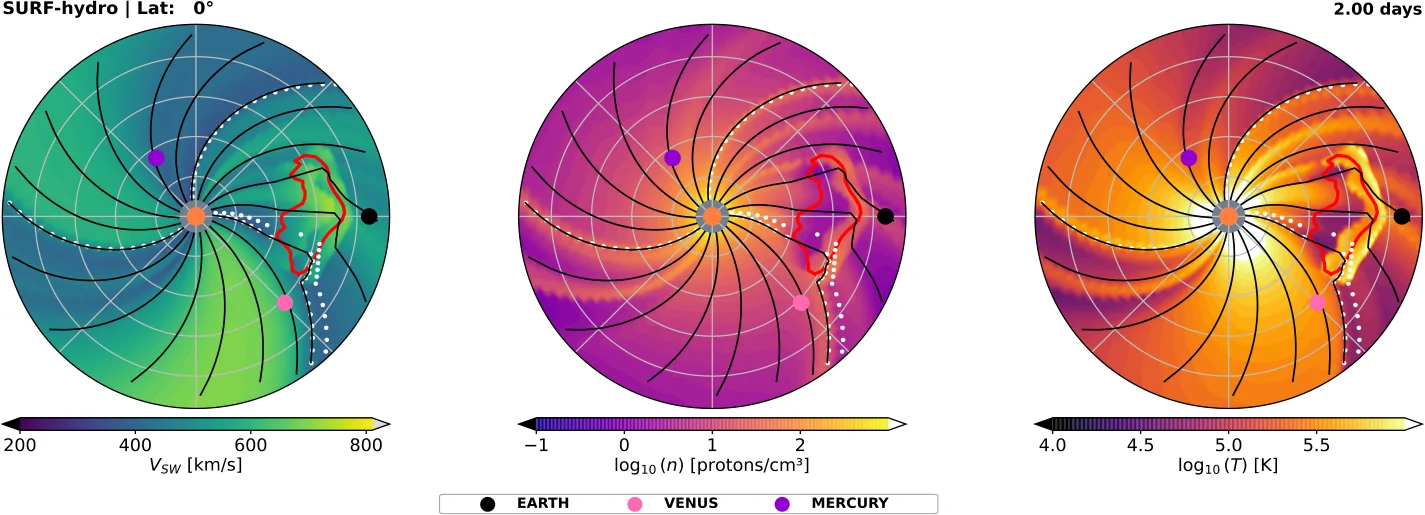
An example of the solar equatorial plane for a SURF-hydro solution for an arbitrary CME (directed at Earth, with angular width of 60^∘^ and speed 800 km s^−1^) into a random WSA solar wind solution of v and $B_{R}$ at 0.1 AU. Ambient density and temperature are determined from Table 2. The CME is represented by a v perturbation only; all other CME parameters are left at ambient values. Panels, from left to right, show snapshots of v, log_10_(n_P_) and log_10_(T), respectively, 2 days after the CME launch. Red lines show the CME boundary, as determined by tracer particles. Black lines show streak lines of the flow, which approximate heliospheric magnetic field lines. White dots show the location of the heliospheric current sheet.

Figure 9 shows an example of typical CME solution in SURF-hydro. It uses a randomly-selected WSA solution to provide ambient solar wind v and $B_{R}$ at 0.1 AU. v at 0.1 AU is reduced using the relations in the first two columns of Table 2. Density and temperature at 0.1 AU are generated by interpolating the remaining values in Table 2. These boundary conditions are used to generate a structured ambient solar wind that contains the heliospheric current sheet (HCS) within the slow wind, shown as the white dashed line in Figure 9. Additional heliospheric magnetic field (HMF) lines are traced as streak lines in the flow, and shown as black lines in all panels of Figure 9. Enhanced density and temperature fronts, aligned with the Parker spiral, are seen where fast wind meets slow (Pizzo 1991).

Into this ambient solar wind, a CME is inserted as a time-dependent speed perturbation at 0.1 AU, beginning at t=0 and lasting for 12 hours (Owens et al. 2025). It has angular width of $60^{\circ}$ and speed of 800 km s^−1^. For this run, the density and temperature in the CME perturbation are left at ambient values, rather than inserting an over-dense blob. Tracer particles are inserted at 0.1 AU at the time that the CME leading and trailing edges pass the inner boundary. This results in the CME boundary shown as a red contour. It can be seen that there is hot, dense plasma ahead of the CME leading edge; this is the CME sheath region bounded by the shock front (Owens et al. 2005; Siscoe and Odstrcil 2008). This compression also propagates back into the CME body. The remainder of the CME body is cooler and less dense than the surrounding ambient wind, due to greater radial expansion of the ambient wind and hence greater adiabatic cooling.

**Figure 10:**
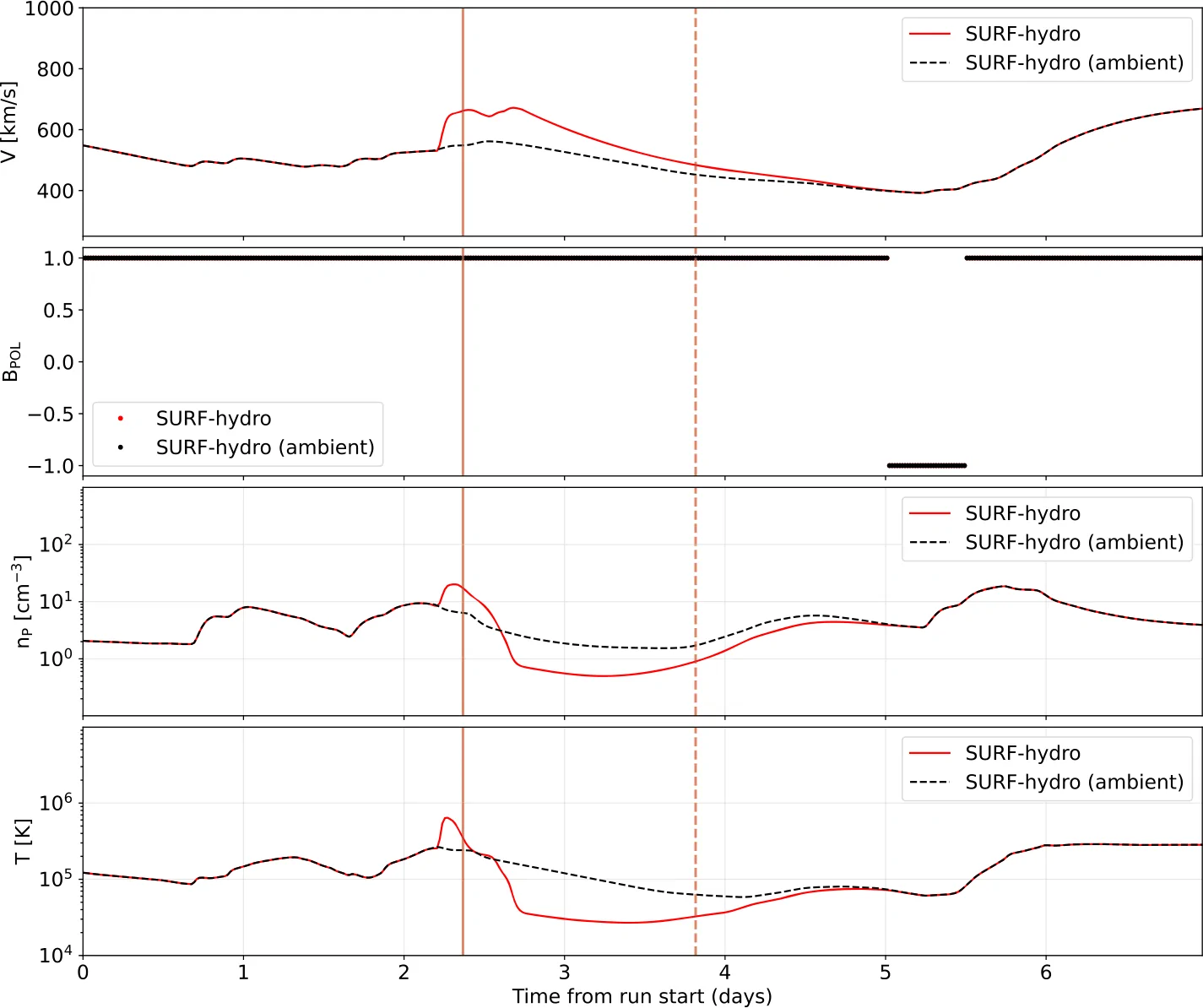
Time series of solar wind properties at Earth for the SURF-hydro solution shown in Figure 9. Red lines show the CME run, black dashed lines show the ambient solar wind run. Panels, from top to bottom, show v, the HMF polarity B_POL_, n_P_ and T. Solid red vertical lines show the time of the CME leading edge, while vertical red dashed lines show the CME trailing edge. These are determined by passive tracer particles in the flow.

Figure 10 shows the resulting time series at Earth, for both an ambient wind run of SURF-hydro (the black dashed lines) and with the CME present (the red solid lines). The vertical lines show the CME leading and trailing edges at Earth determined from the tracer particles. As seen from the snapshot in Figure 9, the interval of solar wind disturbed from the ambient values is greater in extent than the CME perturbation itself. The true time between the shock front and the CME leading edge is only a couple of hours, though if the CME leading edge were to be identified as the time when the density and temperature drops (as appears to be the case with the Richardson and Cane 2010, identifications), it would be misidentified as more than twice that. This longer estimate is in close agreement with the observed typical sheath ahead of an ICME shown in Figure 8, despite the lack of magnetic pressure from compression of the ambient heliospheric magnetic field.

The main body of the CME is characterised by lower density and temperature than in the ambient wind, as observed, along with the characteristic declining velocity profile. These features are indicative of on-going expansion in the radial direction (i.e., beyond the spherical expansion of the ambient solar wind). The CME trailing edge is more difficult to identify from these plasma signatures alone, and the tracer particles show that the CME trailing edge occurs before the return to ambient values. Both the true and apparent CME durations are a little over 24 hours, again in approximate agreement with typical observations (Richardson and Cane 2010, and Figure 8). Thus SURF-hydro reproduces many of the observed features of magnetic cloud CMEs at 1 AU, despite the lack of magnetic pressure forces.

**Figure 11:**
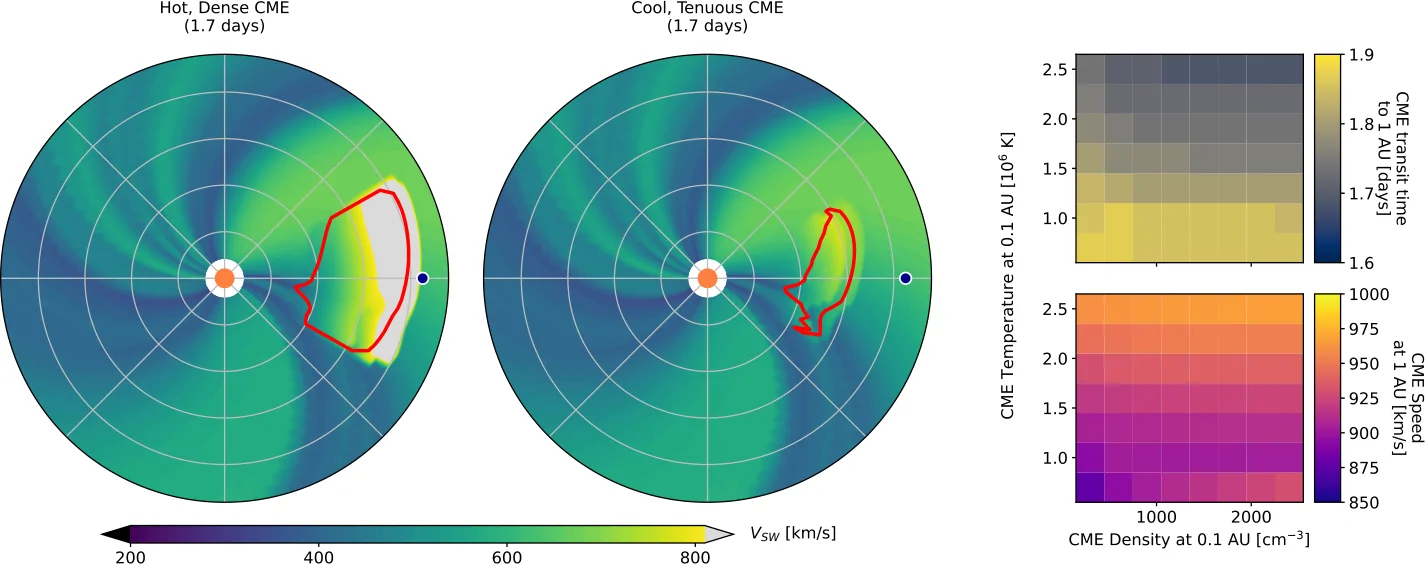
Left and centre: Two SURF-hydro runs after 1.7 days of the same ambient solar wind and with a CME with the same speed, direction and angular width, but different density and temperatures at 0.1 AU. Right: CME arrival time (top) and CME arrival speed (bottom) for a range of initial CME densities and temperatures.

Figure 11 shows a preliminary example of the kind of sensitivity analysis that is made more accessible by SURF-hydro. The two circular plots on the left-hand side show solar wind speed in the solar equatorial plane for very similar SURF runs; the same ambient solar wind, the same CME speed, direction and angular width, but different initial CME densities and temperatures at 0.1 AU. The right-hand plot uses 0.1 AU CME values of $n_{\mathrm{P}} = 2000\text{ cm}^{-3}$ and $T = 2.5 \times 10^{6}\text{ K}$, whereas the middle plot shows a CME with initial values of $n_{\mathrm{P}} = 500\text{ cm}^{-3}$ and $T = 1 \times 10^{6}\text{ K}$. There are significant differences in the resulting solar wind structures. The hot, dense CME arrives at Earth with a shorter transit time and a faster arrival speed, expands more to have greater radial extent and drives a stronger shock with a thicker sheath region.

The two-dimensional colourmaps on the right-hand side of Figure 11 investigate the effect of initial CME density and temperature on the resulting 1-AU transit time and 1-AU arrival speed for this specific ambient solar wind structure. In general, temperature decreases transit time and increases arrival speed, primarily owing to the increased CME expansion. The relation with density is more complex. For higher temperatures, there is a weak negative correlation between transit time and density. This is less prevalent at lower temperature. The key takeaway from this plot is that – for this particular ambient solar wind structure and CME speed – the 1-AU transit time and arrival speed can vary by around 15 – 20%, depending on the initial CME parameters. Thus the initial density and temperature of CMEs merits more study, and ensemble forecasts should likely include perturbations to these highly uncertain, observationally unconstrained parameters.

## Summary and Discussion

In this study, we outlined and tested a one-dimensional, compressible hydrodynamic model of the solar wind. This builds on the previous reduced hydrodynamics “HUXt” model. In order to take advantage of the considerable toolset built up around the HUXt model, we have repackaged the general framework as “SURF”; Space-weather Utilities for Research and Forecasting. HUXt and the new “hydro” models are options within SURF. While hydro is not as computationally efficient as HUXt, it is still rapid enough for use in large ensembles and parametric studies, which can be used to inform and improve future 3DMHD modelling. For example, a single SURF-HUXt 1D run for 5 days of simulation time – adequate to predict CME propagation to Earth – takes around 0.02 seconds on standard desktop CPU. The same run with SURF-hydro is around 0.1 seconds. This is still around $\times 10^{4}$ less computational resource than 3DMHD. And as SURF is written in pure python, it is much simpler to set up and run on standard computer, as well as to integrate within existing forecast and analysis frameworks.

An obvious advantage to solving for density and temperature – rather than just the solar wind flow speed as in HUXt – is that it is possible to use SURF-hydro output to construct synthetic heliospheric imager observations (Eyles et al. 2009; Howard and DeForest 2012; Odstrcil et al. 2020) with relatively low computational overhead. This potentially enables more rigorous data assimilation of heliospheric imager observations (Barnard et al. 2019, 2020, 2023) to improve solar wind reconstructions for both scientific and forecasting use.

Most coronal models provide radial speed and magnetic field as inner boundary conditions to solar wind models (e.g. Arge et al. 2003; Riley, Linker, and Mikic 2001), typically at 0.1 AU. Thus, it is necessary to derive density and temperature from the provided speed. The standard method is to assume some form of equilibrium state at the 0.1 AU inner boundary surface; either constant mass, momentum or kinetic energy flux to determine density, then constant thermal pressure to determine temperature (Pomoell and Poedts 2018). SURF-hydro allows these assumptions to be rigorously tested with comparison to decades of observations. This will form the basis of a future study. Here, however, we have proposed a simple non-equilibrium method to determine inner boundary density and temperature. We use empirical relations derived using 30 years of near-Earth solar wind observations, then “backmap” these relations (allowing for acceleration and expansion) to 0.1 AU. This non-equilibrium method is shown to produce sensible values at 1 AU and to perform favourably in comparison to state-of-the-art methods like WSA-Enlil (Zheng et al. 2013). Having established the effectiveness of this approach with SURF-hydro, this methodology could be easily applied to other solar wind models.

Magnetic cloud CMEs contain the strongest internal magnetic fields, which are not explicitly included in SURF-hydro. Nevertheless, the general features of such CMEs at 1 AU are well captured by SURF-hydro. We find similar CME and sheath durations to those observed, suggesting similar levels of CME expansion and compression of the ambient solar wind. A more detailed comparison will form part of future study.

Properties of CMEs at 0.1 AU are even more uncertain than the ambient solar wind boundary conditions. CME speeds and directions are routinely characterised by coronagraphs (though with unknown, but likely very large uncertainties) and used to define time-dependent boundary condition perturbations in solar wind models (Zhao, Plunkett, and Liu 2002). For operational forecasting, the CME perturbation is assumed to have x4 ambient solar wind density and the same temperature as ambient wind (Taktakishvili et al. 2009). This is in contrast to observations at 1 AU that find CMEs to be cooler and more tenuous than the ambient solar wind, though this is expected to result from in-transit adiabatic expansion (Richardson and Cane 2010). Again, computational limitations mean that these modelling choices have not been systematically explored, something that SURF-hydro can potentially provide in a future study. Here, we show an example of how SURF-hydro can be employed to this issue. For a particular structured solar wind, CME density and temperature at 0.1 AU affects propagation time and arrival speed at Earth by around 15 – 20%. This will be different for different CMEs and ambient solar wind contexts, and could be assessed with suitable perturbations during ensemble forecasting.

## Data Availability

All data are packaged with the analysis code at the Github repository below. WSA-Enlil data were retrieved from https://iswa.ccmc.gsfc.nasa.gov/iswa_data_tree/model/heliosphere/wsa-enlil-cone/, ONMI data wer retrieved from https://omniweb.gsfc.nasa.gov/.

## Materials Availability

N/A

## Code Availability

The SURF code is available from https://github.com/University-of-Reading-Space-Science/SURF. It can also be installed from PyPI or conda-forge. Details can be found at the GitHub URL.
